# COVID-19-related multisystem inflammatory syndrome in adult: the first death in Brazil

**DOI:** 10.1590/S1678-9946202365050

**Published:** 2023-10-09

**Authors:** Maria Lúcia Machado Salomão, Flávia Queiroz, Lina de Moura Mendes, Taiza Maschio de Lima, Murillo de Souza Tuckumantel, Marcia Wakai Catelan, Neymar Elias de Oliveira, Maurício Lacerda Nogueira, Cassia Fernanda Estofolete

**Affiliations:** 1 Faculdade de Medicina de São José do Rio Preto, Departamento de Epidemiologia e Saúde Coletiva, São José do Rio Preto, São Paulo, Brazil; 2 Hospital de Base, São José do Rio Preto, São Paulo, Brazil; 3 Faculdade de Medicina de São José do Rio Preto, São José do Rio Preto, São Paulo, Brazil; 4 Hospital da Criança e Maternidade de São José do Rio Preto, São José do Rio Preto, São Paulo, Brazil; 5 Faculdade de Medicina de São José do Rio Preto, Laboratório de Pesquisas em Virologia, São José do Rio Preto, São Paulo, Brazil; 6 University of Texas Medical Branch, Department of Pathology, Galveston, Texas, United States of America

**Keywords:** SARS-CoV-2, Immune response, Adult multisystem inflammatory disease

## Abstract

The precise pathogenesis of COVID-19-related multisystem inflammatory syndrome remains largely elusive, despite its rarity. The syndrome symptoms often overlap with those of other infections, posing challenges for prompt diagnosis. A male patient, 34 years old, was admitted with suspicion of severe dengue, rapidly progressing to multiple organ dysfunction. Dengue tests resulted negative, and he passed away after four days. This case occurred approximately four weeks after the initial onset of COVID-19 and met all diagnostic criteria as defined by the Centers for Disease Control and Prevention. This report presents the first documented case of fatal multisystem inflammatory syndrome in adult (MIS-A) in Brazil. Recognizing the significance of suspecting this syndrome and promptly initiating treatment at an early stage are essential for minimizing damage and mortality.

## INTRODUCTION

The resulting disease of SARS-CoV-2, known as COVID-19, manifested with a broad spectrum of clinical presentations, ranging from typically flu-like symptoms, such as fever, cough, and fatigue, to severe interstitial pneumonia, acute respiratory distress syndrome, and even multiorgan failure, leading to death^1^. Among several extra-pulmonary complications, one particular syndrome stands out due to its distinct characteristics: the multisystem inflammatory syndrome (MIS). Initially reported in children, the MIS cases have also been observed in adults^2^, leading to its designation as multisystem inflammatory syndrome in adults (MIS-A).

Centers for Disease Control and Prevention (CDC) defines COVID-19-related MIS-A as a severe complication that can occur 2 to 12 weeks after the initial SARS-CoV-2 infection in individuals aged 21 years or older, requiring hospitalization for more than 24 hours or resulting in death. MIS-A diagnosis still involves meeting specific clinical and laboratory criteria. The clinical criteria include having a fever for at least 24 hours prior to hospitalization or within the first three days of it. Additionally, at least three of the primary or secondary clinical criteria should be present before hospitalization or within the first three days of it, and at least one of them must be a primary clinical criterion, such as severe cardiac illness, rash, or non-purulent conjunctivitis. The secondary clinical criteria include new-onset neurologic signs and symptoms; shock or hypotension not explained by medical therapy; abdominal pain, vomiting, or diarrhea; and thrombocytopenia. Regarding laboratory criteria, it requires a recent positive test result for SARS-CoV-2 infection using polymerase chain reaction (PCR), antigen, or antibody testing. Furthermore, elevated levels of at least two of the following markers should be observed: C-reactive protein (CRP), ferritin, interleukin-6 (IL-6), erythrocyte sedimentation rate (ESR), and procalcitonin^3^.

The Brazilian Ministry of Health^4^ aligns with CDC definitions and establishes as a suspected case any patient over 20 years old who requires hospitalization or evolving to death, has been diagnosed with COVID-19, or has had close contact with a COVID-19 case within the last 12 weeks. Additionally, it includes patients presenting: i) fever lasting three days or more; ii) at least two symptom alterations in the systems such as skin alterations, gastrointestinal discomfort (abdominal pain, vomiting, diarrhea), shock or hypotension, neurological symptoms as lethargy, altered mental status, headache, or cardiovascular symptoms including myocarditis, pericarditis, and cardiac failure; and iii) elevated inflammation markers in laboratory tests such as increased CRP, ESR, or ferritin. The case is confirmed when suspected patients present at least two signs of active disease: i) increased brain natriuretic peptide or troponin; ii) increased neutrophils, lymphopenia, and/or thrombocytopenia (<150,000/mm^3^); iii) cardiac involvement by echocardiogram or magnetic resonance imaging including myocarditis and/or pericarditis by electrocardiogram; and iv) rash and/or non-purulent conjunctivitis.

As evident, MIS exhibits a wide range of manifestations, affecting various systems, and accompanied by elevated inflammatory markers. In literature, most cases occurred in males, aged around 30 years, with different outcomes, including deaths^5^. Within this context, we present a report detailing the first documented death from multisystem inflammatory syndrome in an adult patient in Brazil.

## CASE REPORT

On January 17, 2023, a previously healthy 34-year-old man was admitted to the intensive care unit (ICU) of the Hospital de Base de Sao Jose do Rio Preto presenting malaise, daily fever, myalgia, vomiting, abdominal pain, prostration, loss of appetite, fainting, and mental confusion. The onset of his symptom began five days before hospitalization. Two days after the onset of symptoms, he experienced persistent fever, headache, myalgia, arthralgia, and skin rash, leading up to hospital admission.

Upon admission, the patient also exhibited low blood pressure of 94 × 50 mmHg, a heart rate of 120 beats per minute, a respiratory rate of 27 breaths per minute, a body temperature of 37.6 °C, and a peripherical oxygen saturation level of 96% with supplemental support. Given the hypotension, the administration of vasoactive drug, specifically noradrenaline at a dosage of 0.25 mcg/kg/min, was initiated. Initially, dengue fever was suspected but the detection of NS-1 antigen yielded negative results. Empiric antibiotics administration (ceftriaxone 2 g/day and doxycycline 200 mg/day) was also started due to suspicion of sepsis. An ultrasonographic examination revealed a thin layer of perirenal fluid around the right kidney, raising the possibility of acute kidney injury. The chest x-ray did not show any notable abnormalities. Table 1 summarizes all the tests conducted.

**Table 1:** Findings in laboratory tests during patient’s hospitalization.

Test	Collection date						Reference value

	Jan 14	Jan 17	Jan 18	Jan 19	Jan 20	Jan 21	
Hemoglobin (g/dL)	14.3	14.3	12.5	12.2	11.1	10.7	12 to 16 g/dl
Hematocrit (%)	42.0	41.1	35.5	34.8	31.1	28.3	36 to 47 %
Leukocytes (mm^3^)	5,060	7,580	9,410	***11,610***	9,220	10,150	4 to 11 x 10^3^
Neutrophils (mm^3^)	4,210	6,950	8,760	***10,750***	8,340	8,100	
Lymphocytes (mm^3^)	590	410	280	360	400	406	
Platelets (x10^3^/mm^3^)	168	***117***	***148***	155	191	249	150 to 450 x 10^3^/mm^3^
Activated partial thromboplastin time (sec)	-	***46.1***	***48.1***	44.3	36.8	-	25 to 45 sec
INR	-	1.32	1.21	1.21	-	-	0.80 to 1.26
Reactive C protein	-	***22.54***	***16.28***	***11.62***	***7.72***	-	up to 0.50 mg/dL
Interleucin-6 (pg/mL)	-	-	-	-	***122.0***	-	< 3.4 pg/mL
Creatinin (mg/dL)	-	***4.42***	***3.97***	***4.70***	***4.93***	-	0.7 to 1.2 mg/dL
Sodium (mmol/L)	-	***133***	***134***	***128***	***132***	-	136 to 145 mmol/L
Potassium (mmol/L)	-	4.90	4.40	4.30	3.70	-	3.5 to 5.1 mmol/L
Urea (mmol/L)	-	***104***	***108***	***139***	***163***	-	up to 50 mg/dL
Natriuretic peptide type B (pg/mL)	-	-	-	**29,751**	-	-	< 125 pg/mL
Troponin (pg/ml)	-	-	2,017	2,151	2,628	-	<14,000 pg/mL
Creatine kinase (UI/L)	-	486	664	546	-	-	39 to 308 UI/L
Lactic dehydrogenase (U/L)	-	-	-	***1,325***	***1,054***	-	1 to 250 U/L
Aspartate amino transferase (U/L)	-	***278***	***246***	***140***	-	-	15 to 24 U/L
Aspartate alanine transferase (U/L)	-	***319***	***401***	***354***	***272***	-	15 to 24 U/L
ERS^#^ (mm in 60 min)	-	-	-	-	-	-	< 25 mm in 60 min
Ferritin^#^ (ng/mL)	-	-	-	-	-	-	< 2000 ng/mL*
Fibrinogen^#^ (mg/dL)	-	-	-	-	-	-	> 250 mg/dL*
Triglycerides^#^ (mg/dL)	-	-	-	-	-	-	< 132.7 mg/dL*
Urine culture	-	Negative	-	-	-	-	Negative
Blood culture (2 samples)	-	Negative	-	-	-	-	Negative
Anti-leptospirosis IgM^1^		Non reagent					Non reagent
RT-PCR for dengue^2^			Negative				Negative
anti-dengue IgM^3^							
RT-PCR Chikungunya^2^			Negative				Negative
RT-PCR Zika^2^			Negative				Negative
Real time PCR for cytomegalovirus^4^				Negative			Negative
anti-SARS-CoV-2 IgG^5^		Reagent (862,7 AU/ml)					Reagent as ≥ 1 AU/mL
Anti-SARS-CoV-2 IgG^6^		Reagent (4.519)					Reagent > 0.4
Anti-Epstein Baar IgM^7^		Non reagent					Non reagent
Anti-Measles IgM^7^				Non reagent			Non reagent
Anti-Rocky Mountain Spotted Fever IgM^8^			Non reagent (<1/64)				Non reagent (<1/64)

INR = international normalized ratio; RT-PCR = reverse transcriptase polymerase chain reaction; IgM = Immunoglobulin type M; IgG = Immunoglobulin type G; MAC-ELISA = Capture enzyme-linked immunosorbent assay; ^#^Non performed during hospitalization; *Reference value adjusted according to score for hemophagocytic syndrome^13^; ^1^PanBio^TM^ Leptospira IgM ELISA, Abbott Pty., Ltd., Queensland, Australia; ^2^TaqMan^®^ Triplex Kit, Thermo Fisher Scientific Inc., LT, Lituhania; ^3^MAC-ELISA Panbio^TM^, Abbott Pty., Ltd., Queensland, Australia; and MAC-ELISA anti-dengue IgM, CDC, Atlanta, USA; ^4^Cobas 4800 system, Roche Diagnostic International AG, Rotkreuz, Switzerland; ^5^Maglumi^®^ SARS-CoV-2 S-RBD IgG, Snibe Diagnostic, Shenzhen, China; ^6^COVID-19 IgG MAX ELISA, Advagen Biotech, Itu, SP, Brazil; ^7^Chemoluminescence; ^8^Indirect immunofluorescence.

The hospital epidemiological surveillance team, which had been closely monitoring the case, suspected that the patient was suffering from COVID-19-related multisystem inflammatory syndrome. Subsequently, the medical team conducting the investigation on the patient’s COVID-19 history was able to identify a reverse-transcriptase PCR to COVID-19 positive in December 2022. When questioned, the patient reported symptoms such as cough, sore throat, and myalgia, with COVID-19 diagnosis on December 19, 2022.

**Figure 1:**
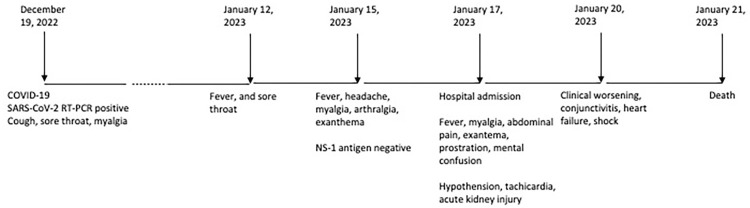
Timeline of signs and symptoms of multisystem inflammatory syndrome in adults case and temporal correlation with acute episode of coronavirus disease 2019.

Three days after ICU admission, his condition began to deteriorate further. He developed worsening skin rash, conjunctivitis, and shock. A transthoracic echocardiogram revealed an ejection fraction of 63% and mid-basal hypokinesia in left and infer-septal wall of left ventricle. In an attempt to manage the condition, the medical team initiated treatment with human immunoglobulin (2 g/kg) and methylprednisolone (2 mg/kg/day). At this moment, the macrophage activation syndrome was also considered, although the patient had been presenting only fever, without hematological abnormalities or organomegalies. Then, other laboratory findings were not investigated. The patient’s condition remained severe and progressed to multiorgan failure. The patient passed away four days after admission, 33 days after COVID-19 diagnosis (Figure 1).

The case was reported to the Brazilian Ministry of Health and, after thorough investigation, it was determined that the event met the criteria for MIS-A, which was temporally associated with COVID-19. This was the first confirmed case of MIS-A resulting in death in Brazil.

This study was submitted and approved by the Research Ethics Committee of the Faculdade de Medicina de Sao Jose do Rio Preto (FAMERP), Sao Paulo, Brazil (protocol Nº 58659522.1.0000.5415, June 10, 2022).

## DISCUSSION

We present the first fatal case of MIS-A in Brazil. The patient has had COVID-19 diagnosis around 30 days before, and at hospital admission it was misdiagnosed as severe dengue. The hospital’s epidemiological surveillance team, which closely monitored the case, suspected that the patient was suffering from COVID-19-related MIS-A. Despite all therapeutic interventions, the patient died in a short time, fulfilling all criteria established by international and Brazilian health authorities.

In May 2020, physicians in the United Kingdom reported a cluster of children presenting fever, cardiovascular shock, and involvement of multiorgan with hyperinflammation. Then, CDC advised surveillance and defined the condition as a multisystem inflammatory syndrome in children (MIS-C) associated with COVID-19^6^. Several months later, the first cases of a similar syndrome in adults, known as MIS-A, started to be reported^2^. In Brazil, until December 10, 2022, 3,395 COVID-19-related MIS-A were reported, being 1,960 confirmed^7^. In this report, we present the first documented death due to MIS-A in Brazil, involving a 34-year-old man.

The Brazilian Ministry of Health^4^ follows the definition of MIS-A provided by CDC and added the following criteria for confirmed case: elevated levels of BNP or NT-proBNP or troponin; increased neutrophil count and reduced lymphocyte count in the blood; platelet count lower than 150,000/mm^3^; cardiac involvement confirmed by echocardiogram or magnetic resonance imaging; and evidence of myocarditis and/or pericarditis on electrocardiogram.

The MIS-A diagnosis presents challenges, particularly due to nonspecific clinical and laboratory findings, as well as overlapping signs and symptoms with other infection, such as dengue or sepsis, like our case. Our patient had fever for more than three days, along with dermatological, neurological, and gastrointestinal symptoms, in addition to hypotension. This clinical picture could have been mistaken for severe dengue. However, the patient presented laboratorial findings indicative of hyperinflammation, which raised suspicion for MIS-A. Furthermore, the presence of myocardial injury, as demonstrated by the echocardiogram, reinforced the MIS-A hypothesis. MIS-A was confirmed considering the patient’s COVID-19 history, which occurred within approximately four weeks after symptoms onset, as observed by the surveillance team.

The exact prevalence of MIS-A remains unknown due to limited data in the form of case reports, potential confusion of its clinical characteristics with other diseases, and presence of fatal outcomes that precedes the definitive diagnosis. Kunal *et al*.^5^ describes 79 cases of MIS-A from 53 scientific articles. Among the cases analyzed, a higher occurrence was observed in males, with a mean age of 31 years. The average duration between onset of MIS-A symptoms and hospitalization raged from 5-8 days, with fever and rash being the most common clinical signs. In another study, Patel *et al*.^8^ points that 221 reports of patients with MIS-A were identified in the global literature from May 1, 2020 to May 25, 2021. The average age of patients was 21 years, with a higher prevalence in males, and no underlying comorbidities in 58% of patients. The most observed symptoms included fever, hypotension, cardiac impairment, and diarrhea, with an average of five organ systems being involved. Such findings closely resemble the profile of our case.

Although lung injury is the most common complication of COVID-19, the disease can also lead to various complications affecting multiple systems, often associated with inflammatory disruption. Such intricate interplay of the inflammatory response during COVID-19 in adults posed initial challenges in comprehending and characterizing cases of MIS-A^9^. Similar to severe COVID-19, the pathogenesis of MIS-A is linked to immune activation and cytokine storm, which involves multiple inflammatory pathways^10^. Moreover, the clinical characteristics of MIS-A and MIS-C are remarkably similar, although reports suggest that the severity of cardiac failure, thrombosis incidents, and mortality may be higher in MIS-A^2,11^. Although COVID-19 and MIS share similarities regarding hyperinflammation and cytokine storm, they present distinct pathological differences. The most notable difference lies in systemic involvement since severe acute respiratory syndrome is commonly associated with COVID-19 and its severity, whereas severe lung injury is uncommon in MIS cases^12^.

Despite the declining incidence of COVID-19 due to global vaccination efforts, continuous monitoring of MIS-C and MIS-A remains crucial to understand the epidemiological profile of these conditions and implement necessary measures. As the number of COVID-19 cases decreases, there is a risk of misinterpreting the clinical findings of these syndromes as symptoms of more prevalent diseases with similar symptoms. However, it is important to always consider MIS-A as a potential diagnosis in the emergency department, especially among patients exhibiting pronounced inflammatory conditions without clear indications of another disease or infection.

Just as the initial cases of acute COVID-19 infection taught us valuable lessons, the adverse outcomes associated with MIS-A should serve as a catalyst for reflection and improvement in terms of prompt and appropriate patient management. It is crucial to raise awareness and suspicion of this condition to ensure early treatment, whether by supportive care or immunomodulation, which can minimize long-term complications and even prevent fatalities.

## References

[1] Wang D, Hu B, Hu C, Zhu F, Liu X, Zhang J (2020). Clinical characteristics of 138 hospitalized patients with 2019 novel coronavirus-infected pneumonia in Wuhan, China. JAMA.

[2] Morris SB, Schwartz NG, Patel P, Abbo L, Beauchamps L, Balan S (2020). Case series of multisystem inflammatory syndrome in adults associated with SARS-CoV-2 infection: United Kingdom and United States, March-August 2020. MMWR Morb Mortal Wkly Rep.

[3] Centers for Disease Control and Prevention Multisystem inflammatory syndrome in adults (MIS-A): case definition and information for healthcare providers.

[4] Brasil, Ministério da Saúde, Secretaria de Vigilância em Saúde, Departamento de Imunização e Doenças Transmissíveis Nota Técnica nº 38/2022-DEIDT/SVS/MS. Atualização acerca da notificação da Síndrome Inflamatória Multissistêmica em Adultos (SIM-A) associada à Covid-19.

[5] Kunal S, Ish P, Sakthivel P, Malhotra N, Gupta K (2022). The emerging threat of multisystem inflammatory syndrome in adults (MIS-A) in COVID-19: a systematic review. Heart Lung.

[6] Centers for Disease Control and Prevention Emergency preparedness and response: multisystem inflammatory syndrome in children (MIS-C) associated with coronavirus disease 2019 (COVID-19).

[7] Brasil, Ministério da Saúde, Secretaria de Vigilância em Saúde (2022). Doença pelo novo coronavírus: COVID-19. Bol Epidemiol.

[8] Patel P, DeCuir J, Abrams J, Campbell AP, Godfred-Cato S, Belay ED (2021). Clinical characteristics of multisystem inflammatory syndrome in adults: a systematic review. JAMA Netw Open.

[9] Ramos-Casals M, Brito-Zerón P, Mariette X (2021). Systemic and organ-specific immune-related manifestations of COVID-19. Nat Rev Rheumatol.

[10] Weatherhead JE, Clark E, Vogel TP, Atmar RL, Kulkarni PA (2020). Inflammatory syndromes associated with SARS-CoV-2 infection: dysregulation of the immune response across the age spectrum. J Clin Invest.

[11] Belay ED, Cato SG, Rao AK, Abrams J, Wilson WW, Lim S (2022). Multisystem inflammatory syndrome in adults after severe acute respiratory syndrome coronavirus 2 (SARS-CoV-2) infection and coronavirus disease 2019 (COVID-19) vaccination. Clin Infect Dis.

[12] Vogel TP, Top KA, Karatzios C, Hilmers DC, Tapia LI, Moceri P (2021). Multisystem inflammatory syndrome in children and adults (MIS-C/A): case definition & guidelines for data collection, analysis, and presentation of immunization safety data. Vaccine.

[13] Fardet L, Galicier L, Lambotte O, Marzac C, Aumont C, Chahwan D (2014). Development and validation of HScore, a score for the diagnosis of reactive hemophagocytic syndrome. Arthritis Rheumatol.

